# Numerical modelling and experimental verification of thermal effects in living cells exposed to high-power pulses of THz radiation

**DOI:** 10.1038/s41598-021-96898-0

**Published:** 2021-09-09

**Authors:** D. S. Sitnikov, A. A. Pronkin, I. V. Ilina, V. A. Revkova, M. A. Konoplyannikov, V. A. Kalsin, V. P. Baklaushev

**Affiliations:** 1 Joint Institute for High Temperatures of the Russian Academy of Sciences, Izhorskaya 13 Bldg. 2, Moscow, Russia 125412; 2 Federal Research and Clinical Center of Specialized Medical Care and Medical Technologies of the Federal Medical and Biological Agency of Russia, Orekhovy Boulevard 28, Moscow, Russia 115682; 3 Institute for Regenerative Medicine, Sechenov First Moscow State Medical University, Trubetskaya str. 8-2, Moscow, 119991 Russia

**Keywords:** Terahertz optics, Biophysics, Thermodynamics

## Abstract

Exposure of cells or biological tissues to high-power pulses of terahertz (THz) radiation leads to changes in a variety of intracellular processes. However, the role of heating effects due to strong absorption of THz radiation by water molecules still stays unclear. In this study, we performed numerical modelling in order to estimate the thermal impact on water of a single THz pulse as well as a series of THz pulses. A finite-element (FE) model that provides numerical solutions for the heat conduction equation is employed to compute the temperature increase. A simple expression for temperature estimation in the center of the spot of THz radiation is presented for given frequency and fluence of the THz pulse. It has been demonstrated that thermal effect is determined by either the average power of radiation or by the fluence of a single THz pulse depending on pulse repetition rate. Human dermal fibroblasts have been exposed to THz pulses (with an energy of $15\,\upmu \hbox {J}$ and repetition rate of 100 Hz) to estimate the thermal effect. Analysis of heat shock proteins expression has demonstrated no statistically significant difference ($p < 0.05$) between control and experimental groups after 3 h of irradiation.

## Introduction

Water, known to be the “matrix of life”^1^, in which chemical and biological processes take place, has attracted enormous attention. Water heating effect by the electric field of electromagnetic radiation has been studied theoretically and experimentally through temperature jumps (T-jump) employing both infrared laser and terahertz (THz) pulsed radiation (see, e.g.^2,3^). THz sources are considered to be more efficient due to direct coupling to the low frequency intermolecular modes of water^4,5^, providing spatially more uniform T-jumps^3^. Unfortunately, temperature estimations obtained for intensities on the order of $1\,\hbox {TW}/\hbox {cm}^2$ are hard to apply to lower intensities of THz radiation typically used for cell exposure in life-science applications. Besides its important contribution to understanding of dynamical properties of water, THz radiation has vast potential in medicine and biology^6–9^. Similar to other bands of the electromagnetic spectrum, the biological effects of THz radiation observed experimentally can be divided into thermal and non-thermal ones (see reviews, e.g.^10–12^). The results obtained thus far in studies of these effects are somewhat controversial^10,13–15^. However, to date, it is clear that irradiation parameters, such as frequency, power density and exposure duration, largely determine whether thermal or non-thermal effects become dominant.

In addition to the direct diagnostic techniques used in life sciences to assess thermal impact of THz radiation (e.g., expression of heat shock proteins^13,16^ and infrared cameras^13,17^), numerical modeling is also actively applied. Temperature increase related to absorption of continuous wave (CW) THz radiation of given power and frequency was initially estimated for water^18,19^; more complicated models were presented for the brain and the breast^20^.

Electric field strength of tightly focused THz radiation of modern high-power sources can reach several tens of MV/cm^21^ or even GV/cm^22^ that facilitates a start of new whorl of the spiral in studying effects of THz radiation on living cells. Development of new high-power pulsed sources of THz radiation (e.g., based on optical rectification) enables one to decrease thermal impact on tissues and cells via reducing the pulse repetition rate while keeping pulse peak power and energy as high as possible and opens up new possibilities in studying the mechanism of interaction of high-power THz pulses with cells. Thermal effect can be reduced to a certain limit determined by temperature increase caused by absorption of a single pulse of THz radiation. The present article is devoted to estimation of temperature increase of water due to the absorption of a single THz pulse and a series of pulses in the course of cell irradiation. In order to do so, we employ a two-dimensional finite element analysis; the temperature dynamics between the successive THz pulses is assessed as well. The proposed model is validated by comparing its results with those obtained via a widely used steady-state model (for CW THz radiation)^18^. Our theoretical estimations of temperature rise have been verified through evaluation of expression levels of heat shock proteins (HSP) in human fibroblasts exposed to THz radiation.

## Results and discussion

### Parameters of THz pulsed radiation

**Figure 1:**
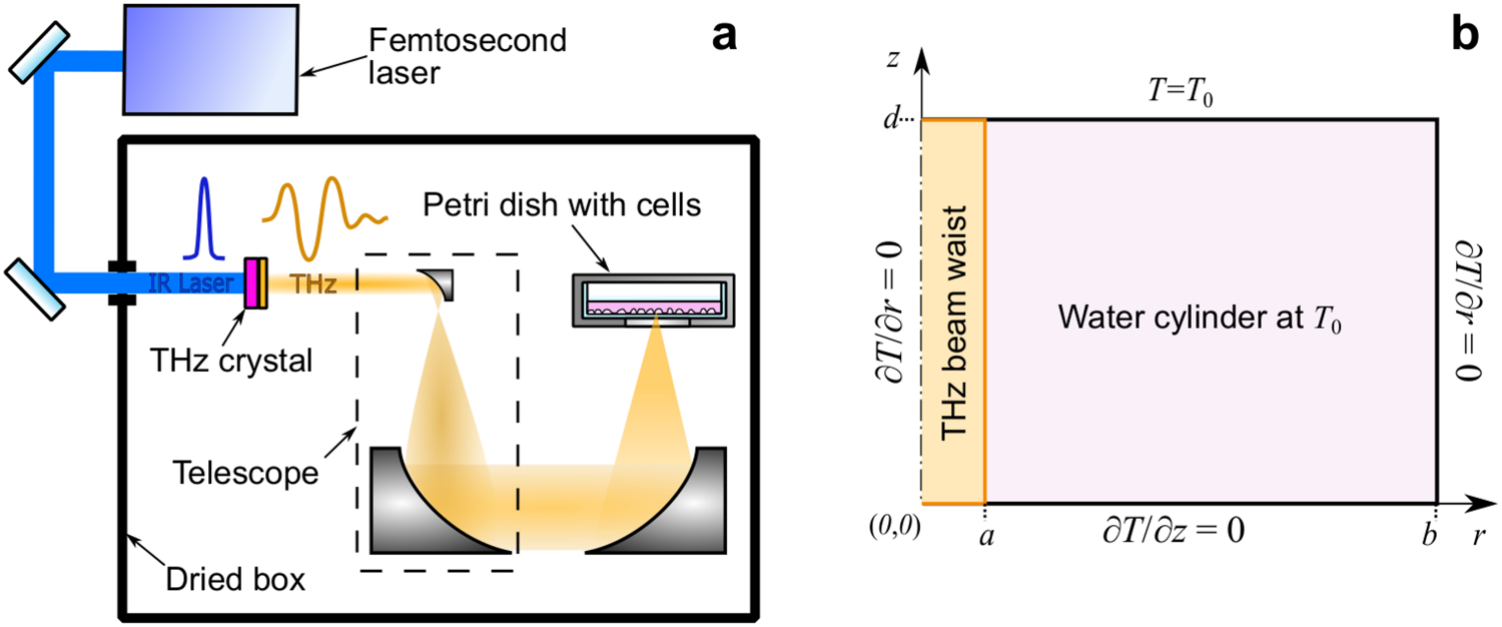
Experimental setup for cell irradiation. (**a**) Schematic diagram. (**b**) Cross-section of the water cylinder used in numerical modelling of heat transfer caused by exposure to THz radiation: *b* and *d* are the radius and the thickness of the cylinder, respectively, *a* is the radius of the THz beam.

Parameters of THz radiation used in numerical model were set equal to those employed in the experimental set-up for living cell exposure^23,24^. More details on the experimental setup for cell irradiation presented in Fig. 1a as well as on the spot size, spectrum, and pulse duration measurements can be found elsewhere^25^. THz pulses with energy $E_{\mathrm{THz}} =18 \pm 0.5$ [$\upmu \hbox {J}$], pulse duration $\tau _{\mathrm{THz}}=484\,[\hbox {fs}]$ at full width half maximum and repetition rate of $f_{\mathrm{p}}=100\,[\hbox {Hz}]$ were obtained through optical rectification in an OH1 [2-(3-(4-hydroxystyryl)-5.5-dimethylcyclo-hex-2-enylidene) malononitrile] organic crystal with conversion efficiency up to 2%^26^. The radiation was focused to a spot with radius $a= 240\,[\upmu \hbox {m}]$ at 1/*e* level and delivered to the cells in the aqueous medium through the bottom of the Petri dish. Taking into account the transmission of the air along the pathway and the plastic dish, the energy, intensity, and fluence of the THz pulse entering the water are $E^{*}_{\mathrm{THz}} =15\,[\upmu \hbox {J}]$, $I_{\mathrm{peak}}^{*}= 30\,[\hbox {GW}\,\hbox {cm}^{-2}]$, and $F^{*}=8.4\,[\hbox {mJ}\,\hbox {cm}^{-2}$], respectively (henceforth $^{*}$ denotes a parameter of the pulse entering the water). In numerical simulations THz radiation directed through the bottom of the Petri dish was presented in the form of a parallel beam of radius *a* in the vicinity of the beam waist (Fig. 1b). For simplicity, the Gaussian intensity distribution of the THz beam was replaced by a flattop beam profile. The simulated area was limited by a cylinder of water of radius *b* and height *d* with an axis coinciding with that of the THz beam.

The THz pulse deposits energy along its path and heats the medium as it passes through. The temperature in the bottom plane of the water cylinder was of practical interest since it should be at a maximum there, resulting in a maximum impact on the cells attached to the bottom of the dish. Assuming that heat is lost mainly through conduction (convection and radiation are considered to be too slow and negligible), the heat diffusion equation, expressed in axisymmetric cylindrical coordinates, may be written as:

1 $$\begin{aligned} \rho \, C_{\mathrm{p}} \frac{\partial T}{\partial t}=\frac{1}{r} \frac{\partial }{\partial r} \left( r\,k(T) \nabla T \right) + \frac{\partial }{\partial z} \left( k(T) \nabla T \right) +S, \end{aligned}$$

where $\rho$ [$\hbox {kg}\,\hbox {m}^{-3}$] is the water density, $C_{\mathrm{p}}$ [$\hbox {J}\,\hbox {K}^{-1}\hbox {kg}^{-1}$] is the specific heat capacity and $k(T)\,[\hbox {W}\,\hbox {m}^{-1}\,\hbox {K}^{-1}$] is the thermal conductivity.

To represent the THz heating power per unit volume the source function *S* [W $\hbox {cm}^{-3}$] in the waist region for a flattop beam profile was used following the formalism of Kristensen et al.^18^:

2 $$\begin{aligned} S = \left\{ \begin{array}{ll} \frac{\alpha \,P}{\pi \,a^2}\,e^{-\alpha \,z}, &{} \text {for}\;0 \leqslant r \leqslant a\\ 0, &{} \text {otherwise}\\ \end{array} \right. , \end{aligned}$$

where $\alpha$ is the absorption coefficient, *P* is the power of the source. Initial and boundary conditions specified for the Eq. (1) are depicted in Fig.1b and detailed in “Methods” section.

The temperature dependence (from $0\,^\circ \hbox {C}$ to $100\,^\circ \hbox {C}$) of the thermal conductivity of water was given by a second-order polynomial^27^:

3 $$\begin{aligned} k(T)=0.6065\left( -1.48445+\frac{4.12292}{298.15}T - \frac{1.63866}{298.15^2}T^2 \right) . \end{aligned}$$

Absorption coefficient $\alpha$ [$\hbox {m}^{-1}$] can be derived from the equations for complex permittivity ($\varepsilon (\nu ,T)$^28^) using the following expression^18^:

4 $$\begin{aligned} \alpha (\nu , T)=\frac{4\pi \nu }{c_{0}}\left[ \frac{\sqrt{\varepsilon '(\nu , T)^2 + \varepsilon ''(\nu , T)^2}}{2}- \frac{\varepsilon '(\nu , T)}{2}\right] ^{0.5}, \end{aligned}$$

where $c_0$ is the speed of light in a vacuum, and $\nu$ is the frequency of THz radiation. For simplicity, we consider THz radiation to be monochromatic at a frequency $\nu =1.5\,[\hbox {THz}]$, which corresponds to the central frequency on the pulse spectrum.. To derive the temperature as a function of time and position, a two-dimensional finite element analysis (MATLAB R2016b) was applied to solve Eqs. (1)–(4).

### Temperature increase resulting from a single THz pulse

As far as not only spatial and temporal distributions of the temperature in water induced by a single pulse of THz radiation are of practical interest, but also temperature changes caused by a series of THz pulses, the heat conduction equation has been solved sequentially for two time domains in our numerical model (hereafter called the *pulse model*). The duration of the first time domain (hereafter called the heating step) is associated with the duration of the THz pulse. For simplicity, the Gaussian envelop of the latter is replaced by a rectangularly shaped pulse with the same energy. The source is turned on, and a given power *P* is kept constant for a specified time $t_{\mathrm{h}}=\tau _{\mathrm{THz}}\,\sqrt{\pi } / \sqrt{4\ln {2}}=515$ [fs]. The power P is set to $P=P^{*}_{\mathrm{peak}}=E_{\mathrm{THz}}^* / t_{\mathrm{h}}=30$ [GW]. The residual temperature distribution in the thin water layer at the end of this time span (just before the start of the next THz pulse) is then calculated, thus setting the initial conditions for the second calculation step (called the cooling step), during which the heat diffusion to the adjacent area and residual temperature are estimated. This step lasts for time $t_{\mathrm{c}} = 1 / f_{\mathrm{p}} - t_{\mathrm{h}} \simeq 0.01$ [s], that is, until the next pulse of THz radiation comes; the pulse repetition rate is $f_{\mathrm{p}}=100\,[\hbox {Hz}]$.

**Figure 2:**
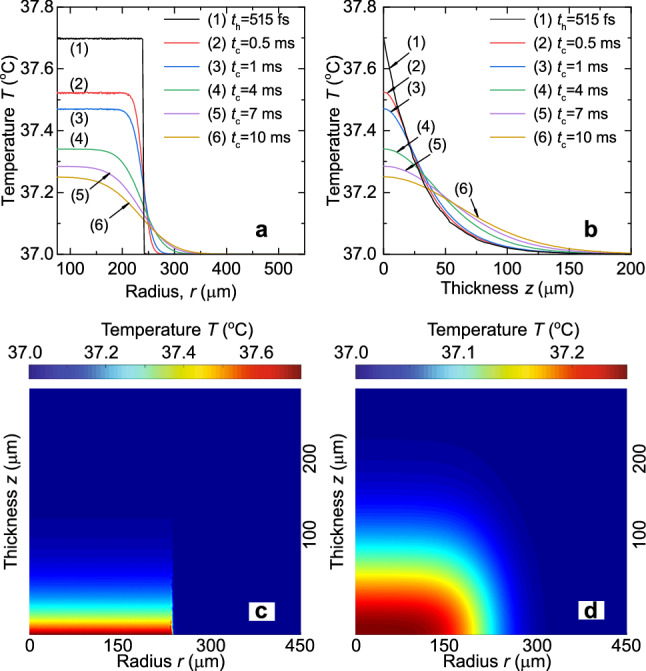
Temperature distributions of water for a single THz pulse. (**a**) Radial (at $z=0$) and (**b**) axial (at $r=0$) temperature dependencies at various time instants. Spatial temperature distribution (**c**) at the end of the THz pulse ($E^*_{\mathrm{THz}}=15\,[\upmu \hbox {J}]$, $t_{\mathrm{h}}=515\,[\hbox {fs}]$) and (**d**) when the next THz pulse comes ($t_{\mathrm{c}}=10\,[\hbox {ms}]$ for $f_{\mathrm{p}}=100\,[\hbox {Hz}]$).

Since the duration of the heating step is negligible compared to that of the cooling step and is not long enough for the heat to diffuse, a step-like temperature increase within the area limited by the beam radius *a* is observed (curve (1) in Fig. 2a). Due to the exponential absorption profile, the solution also behaves exponentially along the *z*-axis, and the THz radiation is fully absorbed within a water layer on the order of 150 $\upmu$m deep (curve (1) in Fig. 2b). Temperature increase at the bottom of the dish at $t_{\mathrm{h}}=515$ [fs] does not exceed $\Delta T(0,0) = 0.7\,^\circ$C (henceforth numbers in brackets like (0, 0) denote the coordinates of a point, here $r=0$ and $z=0$). The spatial distribution of the temperature at the end of the THz pulse is presented in Fig. 2c.

As can be seen from Fig. 2a, the steep profile in the radial direction in the cooling step becomes smoother due to heat diffusion to the adjacent area. The thermal diffusivity $K=k / (\rho \,C_{\mathrm{p}})$ changes slightly in the temperature range of $37\,^\circ \hbox {C}$–$38\,^\circ \hbox {C}$, where it equals $1.52\times 10^{-7}\,[\hbox {m}^2\,\hbox {s}^{-1}$]. It is this small value of *K* that determines the heat accumulation in the area of THz pulse absorption 10 ms after the end of the THz pulse. Our estimations demonstrate that for such a short time and for the maximum temperature increase of $\sim 0.7\,^\circ \hbox {C}$, the heat transfer is defined mainly by thermal conductivity, thus our assumptions on the negligible roles of both convection and radiation seem reasonable.

### Temperature increase resulting from multiple THz pulses

As can be seen in Fig. 2d, a nonzero residual temperature increase is observed (e.g., $\Delta T(0,0)=0.25\,^\circ$C is kept in the center of the THz spot) at $t_{\mathrm{c}}=10$ [ms], when the next pulse of THz radiation comes, giving us temperature map unlike the initial one with $T_0 (r,z)=37\,^\circ$C. So, heat is gradually accumulated in the irradiated area with each pulse absorbed.

Simple analytical solution of Kirchhoff’s heat equation obtained for CW THz radiation^18^ (hereafter called the *CW model*) is commonly used for temperature estimation in life-science applications^29–31^. Such an approach applied for THz pulsed radiation can result in some discrepancy.

**Figure 3:**
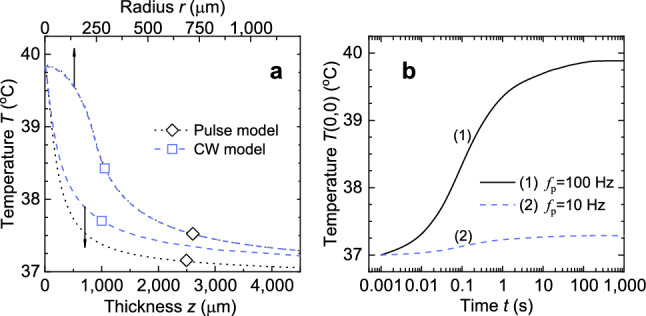
Absorption results for a series of THz pulses. (**a**) Comparison between the radial (at $z=0$) and axial (at $r=0$) temperature distributions for the pulse model (dotted lines) and those obtained for the CW model (dashed lines). (**b**) Temporal evolution of temperature *T*(0, 0) in the beam’s center.

In order to determine the limits of application of the CW model and to verify results of our numerical simulations we performed calculations of temperature increase for a series of pulses with given energy and repetition rate. The CW model operates an average power *P* that equals in our case $P=P_{\mathrm{av}}=E_{\mathrm{THz}}^{*}\,f_{\mathrm{p}}=1.5\,[\hbox {mW}]$. A list of other input parameters includes the following: $N=1000$ is the number of terms to include, $a = 2.4\times 10^{-4}\,[\hbox {m}]$ is the beam radius, $T_0=310.15\,[\hbox {K}]$ is the water initial temperature, $\nu =1.5\,[\hbox {THz}]$ is the frequency of THz radiation, $b = 1.25\times 10^{-2}\,[\hbox {m}]$ and $d = 5\times 10^{-3}\,[\hbox {m}]$ are the radius of the Petri dish and water layer thickness; the rest of the optional parameters are set to their default values. Radial and axial temperature distributions calculated with CW model are presented in Fig. 3a. Temperature increase of water in a steady state reaches $\Delta T(0,0)=2.9\,^\circ \hbox {C}$.

To verify the proposed *pulse model* of water heating, the resulting temperature distribution is compared with that of the CW model; the geometry of the beam and water medium are kept the same. In order to do this the power of the source *S* and the heating duration (pulse duration) in our pulse model are set equal to $P=E_{\mathrm{THz}}^{*}\,f_{\mathrm{p}}=1.5\,[\hbox {mW}]$ and $t_{\mathrm{h}}=1000\,[\hbox {s}]$, respectively. This enables us to trace the dynamics of temperature evolution *T*(0, 0) on its pathway to the steady state (see curve (1) in Fig. 3b). Dotted curves in Fig. 3a demonstrate the cross-sections of the two-dimensional temperature distribution established at $t_{\mathrm{h}}=1000\,[\hbox {s}]$. Good agreement in radial temperature dependence can be observed between the CW model and our calculations. As for axial dependence, our numerical solution goes below the analytical one, making it similar to the results observed by Ganesan et al.^20^.

To minimize the thermal effects, the repetition rate of the THz pulses, and thus the average power of the THz radiation, can be reduced while maintaining pulse energy. Curve (2) in Fig. 3b represents temperature evolution estimated for a series of THz pulses with energy $E^*_{\mathrm{THz}}=15\,[\upmu \hbox {J}]$ and average power $P^*_{\mathrm{av}}=0.15\,[\hbox {mW}]$ for pulse repetition rate $f_{\mathrm{p}}=10\,[\hbox {Hz}]$. Temperature increase obtained in terms of average power gives us $\Delta T=0.29\,^\circ \hbox {C}$ that is lower than $\Delta T=0.7\,^\circ \hbox {C}$ determined by absorption of a single pulse. Thus using CW model for low pulse repetition rates can lead to temperature underestimation.

### Extending the limits of the model application

**Figure 4:**
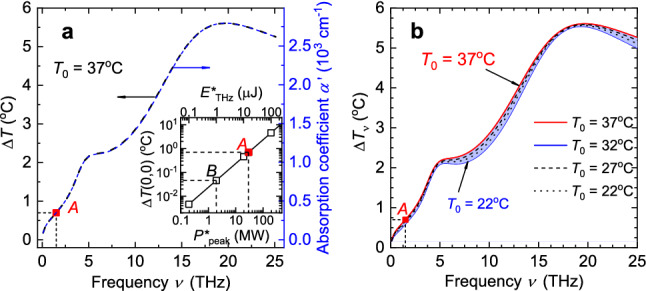
Extending the limits of the FE-model application. (**a**) Temperature increase $\Delta T(0,0)$ in the beam’s center and absorption coefficient $\alpha '$ as a function of radiation frequency $\nu$; $\Delta T(0,0)$ as a function of pulse energy and peak power (inset). (**b**) Temperature increase $\Delta T_{\nu }$ in the beam’s center as a function of frequency for various initial temperatures $T_{0}$. See Data File^32^ for underlying values.

Temperature increase $\Delta T(0,0)$ due to the absorption of a single THz pulse can be scaled to another set of parameters used to describe the THz radiation. According to Eqs. (1) and (2), the heating of the water is proportional to the power *P* of the source *S*. It can be shown that $\Delta T(P^{*}_{\mathrm{peak}})$ linear relationship remains valid with respect to other variables, such as energy $E^{*}_{\mathrm{THz}}$, for certain pulse durations if the heat transfer that occurs during the heating step can be neglected. As a check, we have studied the evolution of the step-like temperature profile obtained at the end of the heating step (curve (1) in Fig. 2a) on pico- to micro-second time scales. Due to the finite mesh size $\Delta r$ used in our calculations, some heat diffusion can be masked within that $\Delta r \approx 0.8\,[\upmu \hbox {m}]$ for small values of $t_{\mathrm{c}}$. Differences in curve shapes become distinguishable only for $t_{\mathrm{c}}>100\,[\hbox {ns}]$ (temperature profiles between $t_{c}=0\,[\hbox {s}]$ and $t_{c}=100\,[\hbox {ns}]$ coincide completely, so data for intermediate $t_{c}$ values is not presented in Supplementary Fig. S1). The same masking of the heat transfer due to conduction up to $t_{\mathrm{c}}>100\,[\hbox {ns}]$ is observed for higher THz pulse energies (up to 0.5 mJ), but the obtained temperature increase is imprecise since convection processes are not taken into consideration. These findings suggest that for THz pulses of durations shorter than 100 ns (typical for most pulsed sources of high power THz radiation, including free-electron lasers and those based on optical rectification and laser-plasma interactions), heat transfer effects can be not taken into account within the duration of the pulse. Thus, the temperature increase in the beam’s center $\Delta T(0,0)$ demonstrates a linear dependence on both the peak power and the energy (see the inset in Fig. 4a).

The curves in Fig. 4a for temperature change $\Delta T(\nu )$ and the reduced absorption coefficient $\alpha '(\nu )=\alpha (\nu )\,\exp [-\alpha (\nu )/p]$, where fitting coefficient $p\approx 2.4\times 10^4\,[\hbox {cm}^{-1}]$, are similar in shape. Dot *A* in the graphs corresponds to the temperature increase calculated for aforementioned experimental parameters (see curves (1) in Fig. 2a,b). All the presented calculations are performed for initial water temperature $T_0=37\,^\circ \hbox {C}$. Since the absorption coefficient $\alpha (\nu ,T_0)$ is a function of two input parameters, a set of curves for various values of $T_0$ is obtained (Fig. 4b). We limit ourselves to a typical room temperature of $T_0=22\,^\circ \hbox {C}$ and the appropriate temperature for a cell culture in an incubator, that is, $T_0=37\,^\circ \hbox {C}$.

For a pulse of THz radiation at frequency $\nu = 1.5\,[\hbox {THz}]$ with energy $E^*_{\mathrm{THz}}=15\,[\upmu \hbox {J}]$ focused to a spot with diameter $480\,\upmu \hbox {m}$, we obtain a temperature increase in the center of the beam equal to $\Delta T(0,0)=0.7\,^\circ \hbox {C}$ for the initial temperature $T_0=37\,^\circ \hbox {C}$, providing us with the temperature change per energy unit of the incident pulse, $\Delta T_{E}=4.6\times 10^{-2}\,[^\circ \hbox {C}\,\mu \hbox {J}^{-1}]$. This temperature increase of $0.046\,^\circ \hbox {C}$ for pulse energy of $1\,\upmu \hbox {J}$ is represented by dot *B* in the inset in Fig. 4a. However, when changing the frequency of the THz radiation, corresponding changes in the spot size due to diffraction should also be taken into account. Strictly speaking, a twofold reduction in the beam size results in a fourfold change in $\Delta T_{E}$. Our estimations have shown that keeping the fluence of the THz pulse $F^*_{\mathrm{THz}}=8.4\,[\hbox {mJ}/\hbox {cm}^2$] constant for various beam radii gives us the same temperature increase $\Delta T$ at the end of the THz pulse (curves for various radii *a* at $t_{\mathrm{c}}=0\,[\hbox {s}]$ are presented in Supplementary Fig. S2). The only differences observed are in the radial temperature profiles at the cooling step for spot size smaller than that considered in our estimations ($a=240\,[\upmu \hbox {m}]$) for instants $t_{\mathrm{c}}>4\,[\hbox {ms}]$. Two conclusions can be made based on the aforementioned results. First, the beam radius does not influence the results of the temperature calculations at the heating step. Second, when calculating the maximum temperature change due to the absorption of the single THz pulse, the fluence value should be considered instead of the energy value, namely, $\Delta T_{F}= 83.3\,[^\circ \hbox {C}\,\hbox {cm}^2\,\hbox {J}^{-1}]$ rather than $\Delta T_{E}$.

So, for a pulse of THz radiation at frequency $\nu$ [THz] with duration $t_{\mathrm{h}}<100\,\hbox {ns}$ having energy density $F^{*}_{\mathrm{Thz}}\,[\hbox {J}\,\hbox {cm}^{-2}]$ and entering the water at temperature $T_{\mathrm{0}}\,[^\circ \hbox {C}]$, the temperature increase $\Delta T\,[^\circ \hbox {C}]$ in the center of the beam ($r=0$, $z=0$) at the end of the pulse can be estimated as:

5 $$\begin{aligned} {\Delta T}=\Delta T'_{\nu }(\nu ,T_{\mathrm{0}})\, \Delta T_{F}\, F^{*}_{\mathrm{Thz}} \simeq \alpha (\nu ,T_{\mathrm{0}})\,\exp [-\alpha (\nu ,T_{\mathrm{0}})/p]\,P(T_{\mathrm{0}}) \, F^{*}_{\mathrm{THz}}, \end{aligned}$$

where $\Delta T'_{\nu }(\nu ,T_{\mathrm{0}})=\Delta T_{\nu }(\nu ,T_{\mathrm{0}})/0.7$ is the reduced dimensionless coefficient derived from $\Delta T_{\nu }(\nu ,T_{\mathrm{0}})$ presented in Fig. 4b, $\alpha (\nu ,T_{\mathrm{0}})\,[\hbox {cm}^{-1}]$ is absorption coefficient, $p=2.263\times 10^4\,[\hbox {cm}^{-1}]$, $P(T_{\mathrm{0}})=b_{0}+b_{1}\,T_{0}+b_{2}\,T^2_{0}$ is a fitting second-order polynomial with coefficients $b_{0}=2.423\times 10^{-3}$, $b_{1}=-5.714\times 10^{-8}$, and $b_{2}=5.037\times 10^{-10}$.

Equation (5) enables us to use either the $\Delta T_{\nu }(\nu ,T_{\mathrm{0}})$ for the exact value of induced temperature change $\Delta T$ (in Dataset, Ref.^32^ values for $\Delta T_{\nu }(\nu ,T_{\mathrm{0}})$ are presented for $T_{0}=22\,^\circ \hbox {C}$, 27$\,^\circ$C, 32$\,^\circ$C, and 37$\,^\circ$C) or $\alpha (\nu ,T_{\mathrm{0}})$ from Eq. (4) and the $P(T_{\mathrm{0}})$ polynomial for arbitrary $T_{0}$. Use of polynomial provides a tolerance of approximation no worse than 1.5% for $T_{\mathrm{0}}$ ranging between 22 and $37\,^\circ \hbox {C}$, as we show in Code (see Ref.^33^) for the right side of Eq. (5).

It should be noted that chemical composition of a cell is rather complex and differs from pure water. Components of a cell in the percentage of total cell weight, are the following: 70% of water, 18% of various proteins, 5% of lipids (the major component of the cell membrane), 2% of polysaccharides and a very small amount of organelles^34^. This suggests that cell thermal properties might differ from that of water. This issue is of great importance and has been addressed previously. Park et al. have shown that thermal conductivity of cells is very close to that of water, but may differ for various cell types. The measured thermal conductivities of HeLa, NIH-3T3 J2, and hepatocyte were $0.6046 \pm 0.018$, $0.5796 \pm 0.017$, and $0.5756 \pm 0.017$ [$\hbox {W}\,\hbox {m}^{-1}\,\hbox {K}^{-1}$] respectively^35^. A later study of Sotoma et al. revealed much lower values of intracellular thermal conductivity $k_{\mathrm{cell}} = 0.11\,[\hbox {W}\,\hbox {m}^{-1}\,\hbox {K}^{-1}]$ using a heater-thermometer hybrid diamond nanosensor^36^. The authors explain this difference by the need to place the heater and detector inside the cell. Sotoma et al. note that thermal conductivity is not a constant and can deviate from its average value. This variation is the greater, the smaller the region where the thermal conductivity is measured. The reason is assumed to be in the complexity of architecture and composition of the intracellular environment. For this reason, we believe that the values of $0.11\,[\hbox {W}\,\hbox {m}^{-1}\,\hbox {K}^{-1}$] should be taken into account when studying the processes of heat transfer inside the cell, instead of the macro-task when the cell layer is surrounded by water.

Due to the short duration of the heating step, changes in the thermal conductivity have tiny influence on $\Delta T$. Temperature increase predicted by our pulse model for cell thermal conductivity $k_{\mathrm{cell}} = 0.5756\,[\hbox {W}\,\hbox {m}^{-1}\,\hbox {K}^{-1}$]^35^ taken for the entire volume of medium equals 0.7 K. The variations in $k_{\mathrm{cell}}$ have a greater effect on the residual temperature at the end of the cooling step by the time the next THz pulse arrives and thus on heat accumulation in the irradiated area. A change in the absorption coefficient $\alpha$ in Eq. (5) due to the difference in the chemical composition of the cytosol and water is expected to have much larger influence on the temperature increase $\Delta T$. Unfortunately, we failed to find the values of absorption of the cytosol at frequency $\nu =1.5\,[\hbox {THz}]$ to make direct comparison with our calculations. However, in his experiments Zou et al. has shown that bulk water demonstrates higher absorption than live cell monolayer^37^. This suggests that our theoretical predictions of temperature increase $\Delta T$ should be considered as an upper limit in case of cell monolayer exposure to THz radiation. Data on complex permittivity of human breast epithelial cells (MCF10A cell line) for $\nu =0{-}1$ [THz] frequency range can be found elsewhere^37^.

The accuracy of assessing the temperature increase in cells exposed to THz radiation can be improved by introducing into the model a thin layer of a medium, which emulates the properties (the thermal conductivity and the absorption coefficient) of cell monolayer. However, this task requires knowing both the true value of the thermal conductivity of the cell cytosol and its absorption in the THz region. This challenge is beyond the scope of this work and will be addressed in our future studies.

### Thermal impact on living cells

Over the past decade, a lot of efforts have been made to explore various techniques for the measurement of intracellular temperature. The existing techniques can be divided into two groups depending on the sensing mechanism:^38^ the first one is based on thermal sensitive fluorescent materials for non-contact measurements, the second one involves the application of contact thermometers. A huge variety of fluorescent thermometers enabling intracellular thermometry (e.g. organic polymer-based or genetically encoded fluorescent thermosensors and inorganic nanoparticles thermosensors) are described in details in Ref.^39^. Fluorescence imaging has the potential to be a powerful method of intracellular thermometry owing to its high spatiotemporal resolution. However, it should be noted that the intracellular environment may affect the optical properties of the fluorescent nano-materials thus leading to unexpected measurement errors and controversial arguments. Contact methods for local temperature measurements usually include various micro-thermocouples^38,40,41^. Compared to fluorescent thermometers, thermocouple probes have a relatively larger size, but they provide more accurate temperature resolution. When choosing an appropriate technique for intracellular thermometry we have taken into account that the methods listed above commonly require an assembly of additional measuring set-up and may be difficult to integrate into the existing experimental set-up for THz irradiation of cells.

As far as cells possess various systems to detect environmental temperature changes, we decided to evaluate cell response to possible thermal stress due to exposure to high-power THz pulses by analysing the level of heat shock proteins (HSPs) expression instead of direct temperature measurement in cells. It is well-known, that HSPs are key proteins that tend to be overexpressed in response to a huge variety of stressors^42^. They can be of physical or chemical nature^43^ such as heat, UV radiation, compression, shearing and stretching, hypoxia, pH shift, nutrient deprivation, or exposure to reactive oxygen species, metals or even alcohols^44^. HSP70 (70-kd protein) is one of the most consistently inducible and highly conserved polypeptides present in all the major intracellular organelles of eukaryotic cells^45^. Stress-induced upregulation of HSP prevents cell damage and facilitate cellular recovery. In mammals, HSPs are classified into several families based on their molecular size. The HSP70 family of heat shock proteins consists of molecular chaperones of approximately 70 kDa in size and represents one of the most ubiquitous classes of chaperones^46^.

**Figure 5:**
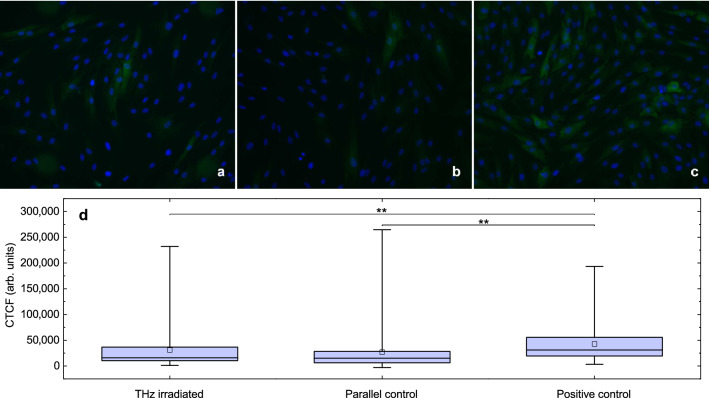
HSPs expression in human fibroblasts. (**a**) Experimental group after exposure to intense pulses of THz radiation for 180 min. (**b**) Control group (no irradiation or external heating). (**c**) Cells after incubation at 40$\,^\circ$C in positive control group, (**d**) Mean fluorescence intensity of secondary goat anti-mouse IgG (H + L) antibodies. $N > 50$ cells/group. Asterisks indicate a statistically significant difference (**$p < 0.05$ by Mann-Whitney t-test).

To estimate HSP70 expression levels in human dermal fibroblasts post THz radiation exposure we have decided to use rather simple and reproducible technique of immunostaining. The immunohistochemistry (IHC) and immunocytochemistry (ICC) are reliable and widely used techniques for the assessment of HSP levels. They have been successfully applied for evaluation of expression levels of HSP to investigate their role in the pathogenesis of cutaneous Lichen planus^47^, psoriasis^48^ or to study the potential of HSP as a biomarker of inflammatory bowel disease^49^ or even as a cancer biomarker^43,50,51^. Human dermal fibroblasts adhered to the Petri dish bottom were exposed to a series of THz pulses with average power $P_{\mathrm{av}}=1.5\,[\hbox {mW}]$ ($E^{*}_{\mathrm{THz}}=15\,[\upmu \hbox {J}]$ and $f_{\mathrm{p}}=100\,[\hbox {Hz}]]$) for 180 min (group 1). Such a time duration usually allows to track the alterations in expression levels of HSP70. Control group cells were kept under the same conditions as experimental ones except exposure to THz radiation (group 2). According to our estimations (see “Model verification” section), local temperature in the center of the THz beam does not exceed $T(0,0)=39.9\,^\circ \hbox {C}$ (for the following set of experimental parameters $E^{*}_{\mathrm{THz}}=15\,[\upmu \hbox {J}]$, $d_{1/e}= 480\,[\upmu \hbox {m}]$, $t_{\mathrm{h}}=180\,[\hbox {min}]$, and $f_{\mathrm{p}}=100\,[\hbox {Hz}])$. So a positive control group of cells heated up to $40\,^\circ \hbox {C}$ in thermoshaker was also prepared for comparison (group 3). The HSP70 expression level in the positive control group differs dramatically from that one of the experimental and control groups (Fig. 5). Corrected total cell fluorescence (CTCF) was measured for each cell. Additional data for intermediate temperatures of positive control groups of cells between $37\,^\circ \hbox {C}$ and $40\,^\circ \hbox {C}$ is presented in Supplementary Fig. S3. A total amount of cells analysed was no less than 50 per group. Normality of fluorescence intensity distribution was checked using Kolmogorov–Smirnov–Lilliefors and Shapiro–Wilk tests. Due to non-normal distribution ($p<0.05$), nonparametric criteria were used for further analysis. Mann-Whitney test demonstrated statistical difference between group 3 and the rest of groups while no difference was observed between groups 1 and 2 ($p<0.05$). Obtained data indicate that our theoretical estimations of upper limit of temperature increase in the center of the THz beam $\Delta T(0,0)=2.9$ [K] look plausible.

## Conclusions

Absorption of THz radiation by water can induce thermal stress in exposed cells. Common approaches for thermal effect estimation in terms of CW power applied a decade ago may failure when a series of high-intensity THz pulses are used. We have shown that temperature increase is determined by either the average power of radiation or by the fluence of a single THz pulse depending on pulse repetition rate. In order to do so, we have developed a FE model for estimating the thermal effects of single THz pulse that can be applied for a variety of sources of THz radiation including free-electron lasers and those based on optical rectification and laser-plasma interactions. We have found that absorption of a single pulse of THz radiation ($I_{\mathrm{peak}}^{*}= 30\,[\hbox {GW}\,\hbox {cm}^{-2}$] and $F^{*}=8.4\,[\hbox {mJ}\,\hbox {cm}^{-2}]$, the most intense to our knowledge applied for living cells irradiation) resulted in the temperature increase of $0.7\,^\circ \hbox {C}$ at the bottom of the dish while exposure to a series of pulses with the same energy $E^{*}_{\mathrm{THz}}=15\,[\upmu \hbox {J}]$ and repetition rate $f_{\mathrm{p}}=100\,[\hbox {Hz}]$ resulted in $2.9\,^\circ \hbox {C}$ of water heating. Since some heat transfer mechanisms (convection and radiation) are excluded in the numerical model, the estimations presented here represent the worst-case scenario in terms of thermal load. Thermal effect of pulses of THz radiation with aforementioned parameters has been studied on human skin fibroblasts. No significant differences have been observed between HSP expression level in cells exposed to THz radiation and control group cells. The results obtained would appear to be important for researchers studying thermal and non-thermal effects in cells at THz facilities when direct measurements of temperature change are not possible.

## Methods

### Initial and boundary conditions for heat transfer equation

The upper surface of the dish bottom is considered to be a zero-point, $z = 0$. Since the setup is designed for cell irradiation and a Petri dish with the cells is mounted on a thermally stabilized heating plate, the initial temperature of the medium $T_0$ at $t=0\,[\hbox {s}]$ is set to $37\,^\circ \hbox {C}$ in the model. The upper side of the cylinder is a water-air interface, maintained at a constant temperature, $T(z=d)=T_{0}$, representing the first-type boundary condition. As for the side walls and the bottom of the dish, water-plastic interface is considered to be adiabatic:

6 $$\begin{aligned} \frac{\partial T}{\partial r} \bigg |_{r=b} = 0,\qquad \frac{\partial T}{\partial z} \bigg |_{z=0} = 0, \end{aligned}$$

since for short calculation time domains $t_{\mathrm{h}}$ and $t_{\mathrm{c}}$ the plastic wall serve as an insulator and do not let the heat leave the water medium. Water absorption $\alpha =353.26\,[\hbox {cm}^{-1}]$ at the initial temperature $T_0=37\,^\circ$C is an order of magnitude higher than that of a plastic dish ($\alpha \sim 14\,[\hbox {cm}^{-1}]$) made of polystyrene^52^, which makes thermal input of the plastic itself negligible. Since we consider axisymmetric geometry, the axis ($r=0$) is also considered adiabatic:

7 $$\begin{aligned} \frac{\partial T}{\partial r} \bigg |_{r=0} = 0. \end{aligned}$$

### Model verification

To verify our model of water being heated by a pulse of THz radiation (pulse model), the resulting temperature distribution is compared with that obtained analytically^18^ for CW THz radiation (hereafter called the CW model). To carry out the comparison, the power of the source *S* and the heating duration (pulse duration) in our pulse model are set equal to $P=E_{\mathrm{THz}}^{*}\,f_{\mathrm{p}}=1.5\,[\hbox {mW}]$ (according to the parameters specified in “Parameters of THz pulsed radiation” section) and $t_{\mathrm{h}}=1000\,[\hbox {s}]$, respectively. As for the CW model, the input parameters are as follows: number of terms included $N=1000$, $P=1.5\,[\hbox {mW}]$, $a = 2.4\times 10^{-4}\,[\hbox {m}]$, $T_0=310.15\,[\hbox {K}]$, $\nu =1.5\,[\hbox {THz}]$, $b = 5\times 10^{-2}\,[\hbox {m}]$, and $d = 1.5\times 10^{-2}\,[\hbox {m}]$, with the rest of the optional parameters being set to their default values.

The temperature *T* in the center of the beam ($r=0$) at the bottom of the water cylinder ($z=0$) (henceforth denoted by *T*(0, 0)) as a function of time is presented in Fig. 3a. It can be seen that the water approaches a steady state after about 250 s. Figure 3b demonstrates the cross-sections of the two-dimensional temperature distribution established at $t_{\mathrm{h}}=250$ [s]. Good agreement in radial temperature dependence can be observed between the CW model (dashed line) and our calculations (dotted line). As for axial dependence, our numerical solution goes below the analytical one, making it similar to the results observed by Ganesan et al.^20^.

### Primary culture of fibroblasts

This study was conducted in accordance with the Declaration of Helsinki and GCP guidelines and was approved by the local Ethical Committee of the Federal Research and Clinical Center of Specialized Medical Care and Medical Technologies (protocol No. 4/5 from December 2, 2019). The patient signed an informed consent before enrolling in this study.

The extraction and the cultivation of the dermal skin fibroblasts were carried out as described previously^24,53^. Briefly, $2\times 1\times 1\,\hbox {mm}$ biopsy specimens were washed in DPBS supplemented with 1X antibiotic-antimycotic solution (Gibco, USA). Then, the dermis was washed with DMEM/F12, mechanically minced, and dissociated in a solution of collagenase I (Gibco, USA) (1 mg/ml) in the DMEM/F12 culture medium with a glucose concentration of 1 g/L. The preparation was incubated for 2 h using an orbital shaker with constant shaking at a temperature of $37\,^\circ \hbox {C}$. It was then washed from the enzyme with the culture medium, passed through a filter ($100\,\upmu \hbox {m}$; SPL Life Science, Korea) to remove the fibers and precipitated by centrifugation. The cell pellet was suspended in the complete DMEM/F12 (Gibco, USA) growth medium containing 1 g/L glucose, 10% fetal bovine serum, 1X GlutaMAX solution, and 1X antibiotic-antimycotic solution, and seeded in a culture flask (Eppendorf), based on the coverage of $1\times 10^6$ cells/$\hbox {cm}^2$ and cultured in a $\hbox {CO}_2$ incubator at 5% $\hbox {CO}_2$ at $37\,^\circ \hbox {C}$ until the 100% confluence of the monolayer was reached, then the cells were dissociated with 0.25% trypsin-EDTA (Gibco, USA). Cells were seeded in the $\mu$-dish with 4 well silicone insert and polymer coverslip (#80466 ibidi, USA) 24 h prior to THz exposure. Right before the experiment, culture medium was changed to DMEM/F12 growth medium containing 1 g/L glucose, 10% fetal bovine serum, 1X GlutaMAX solution, 1X antibiotic-antimycotic solution and 15 mM HEPES (all from Gibco, USA).

### Heat shock proteins analysis

The cells were exposed to pulsed THz radiation for 180 min and fixed immediately after exposure in Petri dish with 4% paraformaldehyde solution contain and 0.1% saponin in PBS (pH 7.4) for 20 min at room temperature, followed by two rinses in PBS and permeabilization with 0.5% Triton-X100 and 0.5% Tween 20 (in PBS, pH 7.4), supplemented by 1% goat serum to block non-specific antibody binding. Cells in positive control group were heated up to $40\,^\circ \hbox {C}$ for 60 min in a thermoshaker (SkyLine SK-3L) in standard culture medium with 15 mM HEPES solution and fixed immediately after the procedure. The fixed-permeabilized cells were then incubated for 1 h at $37\,^\circ \hbox {C}$ with a primary mouse monoclonal antibody against HSP70 (dilution 2:100, MS-482-B1, Lab Vision, ThermoScientific, USA). After several rinses with PBS, the cells were incubated for 1 h with secondary goat anti-mouse IgG (H+L) antibodies (Alexa Fluor 488-conjugated dilution 1:400; Invitrogen USA). Then, the Petri dishes were rinsed several times with DPBS. Hoechst 33342 (Thermo Fisher Scientific) was applied for cell nuclei staining. Immunofluorescence was performed using a CELENA® S Digital Imaging System (Logos, Korea). Fluorescence of stained cells was measured using ImageJ software (ver. 1.53). Image was split into color channels, then contour of each cell was delimited using freehand tool. Area, integrated density and mean grey values were measured for each cell in the image and the background as well. Corrected total cell fluorescence (CTCF) was calculated as CTCF $=$ Integrated Density - (Area of selected cell $\times$ Mean grey value of background readings) for each cell, the total amount of which exceeded 50 in each group. Normality of fluorescence intensity distribution was checked using Kolmogorov-Smirnov-Lilliefors and Shapiro-Wilk tests. Due to non-normal distribution ($p<0.05$), Mann-Whitney test was used for further analysis.

## Supplementary Information

Supplementary Figures.

## Data Availability

The data presented in this manuscript are tabulated in the main paper, in the supplementary materials, and data repository.
