# Supplementary material for: Numerical modelling and experimental verification of thermal effects in living cells exposed to high-power pulses of THz radiation
Source: Sci Rep. 2021 Sep 9;11:17916. doi: 10.1038/s41598-021-96898-0 (PMC8429778; doi:10.1038/s41598-021-96898-0)
Supplement: Supplementary file 1 — Supplementary Figures. [file 41598_2021_96898_MOESM1_ESM.pdf]

Numerical modelling and experimental verification of thermal effects in living cells exposed to high-power pulses of THz radiation

Sitnikov D.S., Pronkin A.A., Ilina I.V., Revkova V.A., Konoplyannikov M.A., Kalsin V.A., and Baklaushev V.P.

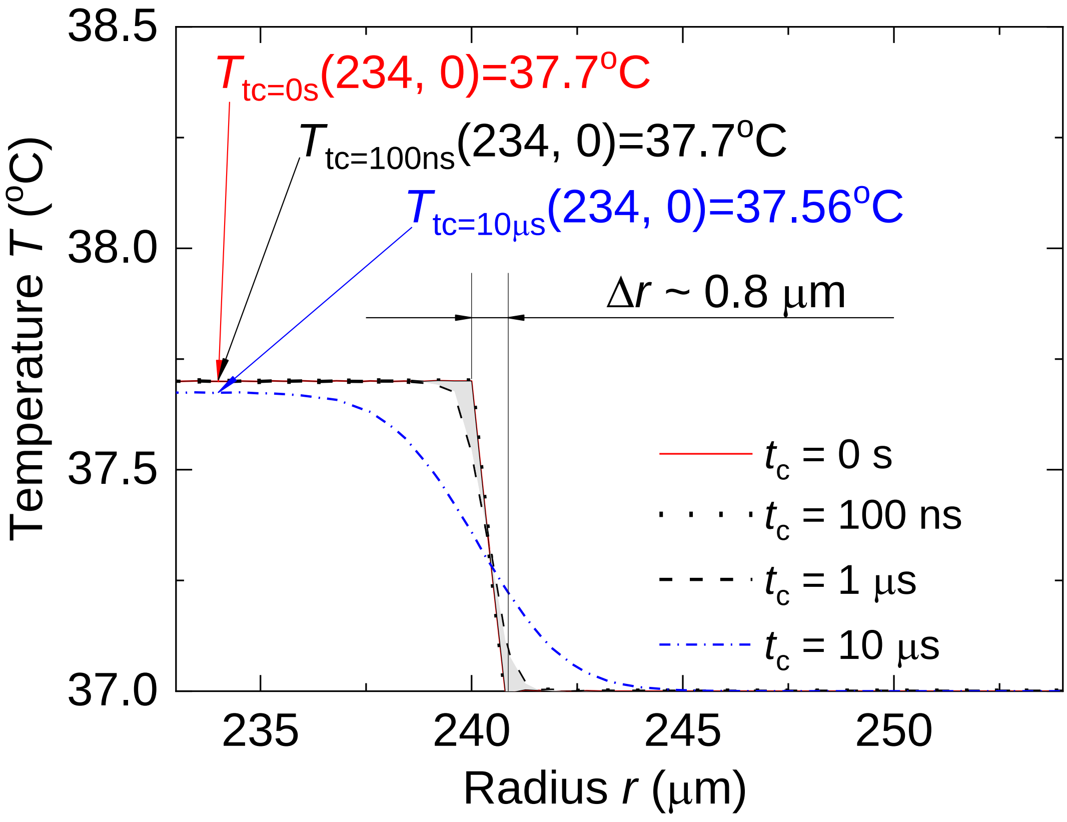

Fig.S1. Dynamics of radial temperature profile $T(r, 0)$ at the cooling step. Temperature values for a point with coordinates ($r = 234$, $z = 0$) in microns are presented.

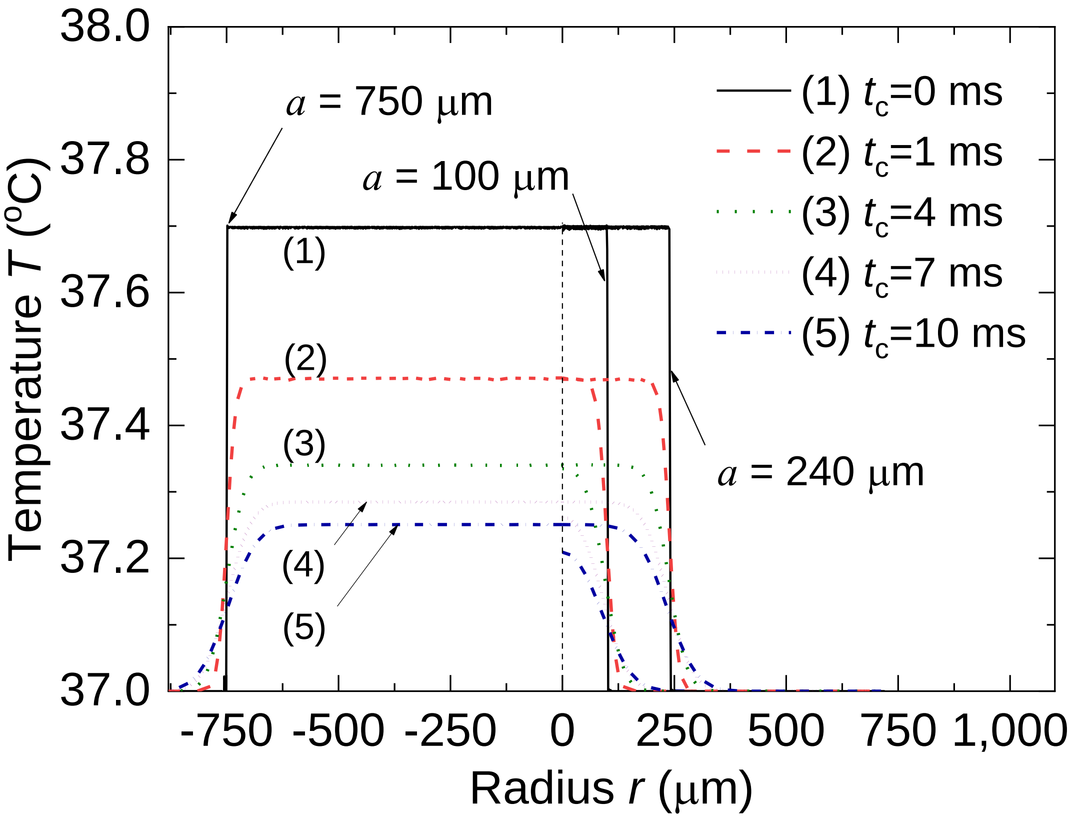

Fig.S2. Temperature profiles $T(r, 0)$ for various beam radii a during the cooling step. Temperatures in the beam center $T(0, 0)$ start to differ for instants $t_c > 4$ ms only (curves (4) and (5)).

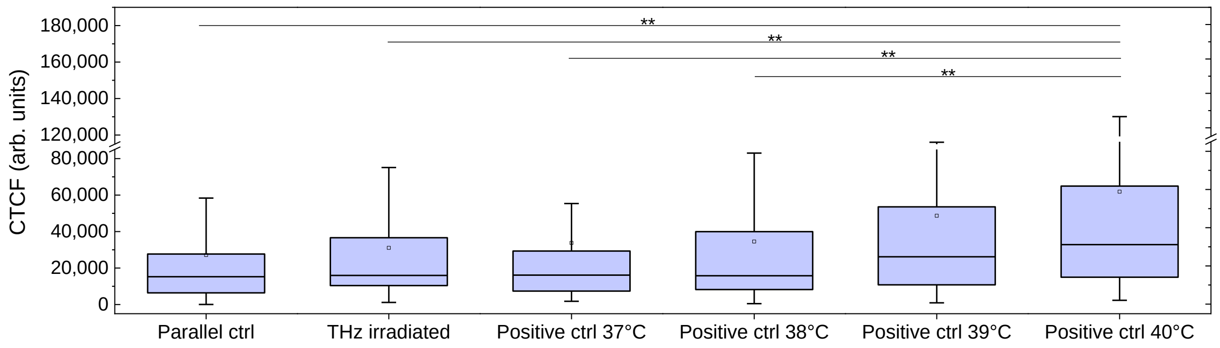

Fig.S3. HSPs expression in human fibroblasts. Data on parallel control and THz irradiated cells groups in comparison with positive control groups. Cells after incubation at 37˚C, 38˚C, 39˚C, and 40˚C in positive control groups. Mean fluorescence intensity of secondary goat anti-mouse IgG (H+L) antibodies. $N > 50$ cells/group. Asterisks indicate a statistically significant difference (**$p < 0.05$ by Mann-Whitney t-test).
